# The Role of Omega-3 Polyunsaturated Fatty Acids in Stroke

**DOI:** 10.1155/2016/6906712

**Published:** 2016-06-28

**Authors:** Jiyuan Bu, Yang Dou, Xiaodi Tian, Zhong Wang, Gang Chen

**Affiliations:** Department of Neurosurgery & Brain and Nerve Research Laboratory, The First Affiliated Hospital of Soochow University, 188 Shizi Street, Suzhou 215006, China

## Abstract

Stroke is the third commonest cause of death following cardiovascular diseases and cancer. In particular, in recent years, the morbidity and mortality of stroke keep remarkable growing. However, stroke still captures people attention far less than cardiovascular diseases and cancer. Past studies have shown that oxidative stress and inflammation play crucial roles in the progress of cerebral injury induced by stroke. Evidence is accumulating that the dietary supplementation of fish oil exhibits beneficial effects on several diseases, such as cardiovascular diseases, metabolic diseases, and cancer. Omega-3 polyunsaturated fatty acids (n-3 PUFAs), the major component of fish oil, have been found against oxidative stress and inflammation in cardiovascular diseases. And the potential of n-3 PUFAs in stroke treatment is attracting more and more attention. In this review, we will review the effects of n-3 PUFAs on stroke and mainly focus on the antioxidant and anti-inflammatory effects of n-3 PUFAs.

## 1. Introduction

Stroke, also known as cerebrovascular insult or brain attack, is defined by World Health Organization as “neurological deficit of cerebrovascular cause that persists beyond 24 hours or is interrupted by death within 24 hours” in the 1970s [1]. Stroke was firstly reported in the 2nd millennium BC, and firstly described by Hippocrates. But up till now, the systematic treatment strategy of stroke remains elusive.

In 1946, Hansen and Burr found that the Eskimos who live in Newfoundland rarely suffer from cardiovascular disease [2]. They owe the beneficial effects to the diet of Eskimos, which is rich in fish and seafood. Fish oil begins to capture people's attention. Further studies indicated that the benefit effects of fish oil are mainly mediated by omega-3 polyunsaturated fatty acids (n-3 PUFAs), which are against a range of diseases, including cardiovascular diseases, inflammatory diseases like arthritis, metabolic diseases like type 2 diabetes, and cancer [3].

The aim of this paper is to summarize the research progress of n-3 PUFAs, especially the effects on stroke.

## 2. Subsets, Sources, and Metabolism of n-3 PUFAs

**Figure 1:**
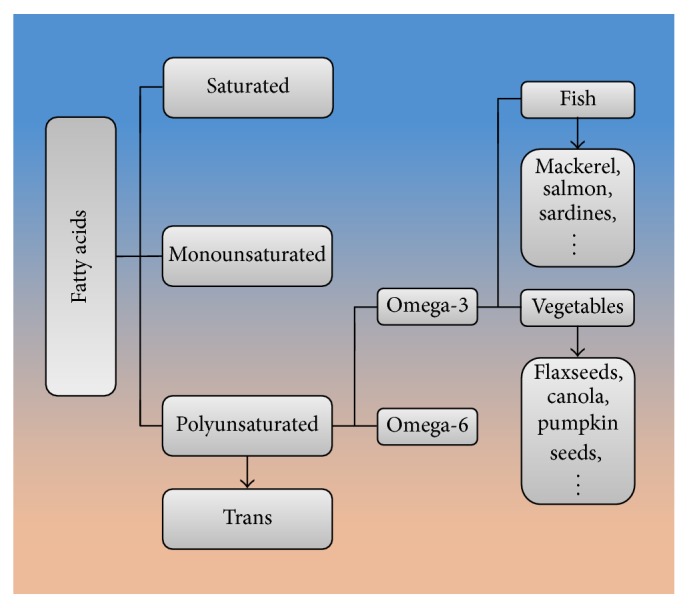
The classification of fatty acids.

According to the number of double bonds in fatty acid side chains, the natural fats are classified into 3 subsets: saturated, monounsaturated, and polyunsaturated. The classification of fatty acids is shown in Figure 1. And there is a fourth artificial subset, trans fats, which is created by hydrogenation [4]. Polyunsaturated fats are further classified into 2 subsets by the first double bond: omega-3 fatty acids and omega-6 fatty acids. n-3 PUFAs have the first double bond at the third carbon from the methyl terminal, whereas omega-6 polyunsaturated fatty acids (n-6 PUFAs) have the first double bond at the sixth carbon [5]. Mammalian cells are short of the desaturase that can convert n-6 to n-3 PUFAs, which means that n-3 PUFAs must be supplied with the diet. Fish, such as mackerel, salmon, sardines, halibut, herring, and tuna, in the human diet is the major source of n-3 PUFAs, containing docosahexaenoic acid (DHA) and eicosapentaenoic acid (EPA). Quite a few kinds of vegetables and vegetable oil, such as flaxseeds, canola, pumpkin seeds, flaxseed oil, canola oil, and perilla seed oil, also can provide n-3 PUFAs, such as alpha-linolenic acid (ALA), which can be converted to EPA and further to DHA by a desaturase enzyme [6]. Isotope-labeled ALA trials suggested that the conversion of natural ALA to EPA is between 0.2% and 21% and further to DHA is between 0% and 9% [7]. The conversion of ALA to DHA and EPA is likely influenced by the competitive inhibition of linoleic acid and negative feedback of DHA and EPA [8]. And the interconversion is limited. So the best way to increase fatty acids intake is to supplement them with specific fatty acids [9].

## 3. Research Tools for n-3 PUFAs

### 3.1. Gas Chromatography Methods

All fatty acids in plasma fraction can be analyzed by high performance liquid chromatography and mass spectrometry.

### 3.2. Fat-1 Transgenic Mice

Mammalian cells are short of the desaturase, which can convert n-6 to n-3 PUFAs [10]. Fat-1 transgenic mice carrying a fat-1 gene expressed a* Caenorhabditis* elegans desaturase that introduces a double bond into n-6 PUFAs to form n-3 PUFAs [11]. Due to the capability, fat-1 transgenic mice are able to produce n-3 PUFAs from n-6 PUFAs and their organs or tissues are rich in n-3 PUFAs without the dietary n-3 PUFAs supplementation [12]. Accordingly, fat-1 transgenic mice can avoid the potential confounding effects from the dietary supplementation [13]. The fat-1 transgenic mice are widely used as new tools for n-3 PUFAs studies.

### 3.3. Administration Pathway

Rats are chosen in most experimental studies, and some studies also use mice, baboons, and piglets as animal models [14]. The common administration way is oral administration or intragastric administration. The dosage of oral drugs is 0.2 g to 30 g of EPA and DHA/kilogram, and the duration of intervention for studies is from 24 h to 4 weeks [15].

## 4. n-3 PUFAs and Stroke

### 4.1. Stroke

Stroke has two main types: ischemic stroke, due to the lack of blood flow, and hemorrhagic stroke, due to the bleeding. Ischemic stroke can be further classified into cerebral infarction and transient ischemic attack (TIA), and hemorrhagic stroke also can be further classified into subarachnoid hemorrhage (SAH) and intracerebral hemorrhage (ICH).

### 4.2. n-3 PUFAs and Ischemic Stroke

Cerebral infarction is defined as the necrosis of the cerebral tissue caused by ischemia. Under normal circumstances, cerebral blood flow (CBF) is 50 ± 10 mL/100 g/min. When CBF drops to 15 mL/100 g/min, cerebral cortical evoked potential and brain waves disappear completely, but cerebral cells are still alive. And when CBF drops to 8–10 mL/100 g/min, even lower, the function of ion pumps in neuron membrane begins to fail, inducing potassium efflux and sodium influx, and cerebral cells begin to die and cerebral infarction occurs. Traditionally, TIA was defined as the episodes of neurologic dysfunction resulting from focal cerebral ischemia and completely recovers within 24 hours [16]. The American Heart Association renewed the definition in 2009 and changed the definition from time-based to tissue-based [15]. The newest diagnosis of TIA is based on the restricted diffusion on MRI [16]. Currently, the diagnosis of TIA is dependent upon CT or MRI findings heavily. Cerebral ischemia/reperfusion (I/R) injury is a phenomenon that ischemic stroke induces cerebral cells damage, and, after the restoration of hemoperfusion, the ischemic injury even becomes more serious.

Early reperfusion is desirable, but reperfusion also induces additional neural tissue injury and the breakdown of cellular integrity by oxidative stress, excitotoxic signaling, inflammation, and others [17]. Elevated oxidative stress is associated with the pathogenesis of cerebral injury in I/R [18]. During cerebral I/R, the endogenous antioxidative defense systems turn to be ineffective, which results from the inactivation of detoxification systems and the degradation of antioxidants [19, 20]. A multitude of oxygen radicals such as reactive oxygen species (ROS) begin to accumulate and cause apoptosis and cellular damage [21]. ROS are involved in the oxidative damage of proteins, nucleic acids, and lipids in ischemic tissues directly [22]. ROS can also cause lipid peroxidation, which leads to the damage of biological membranes [23]. Classic description of lipid peroxidation mainly contains three steps [24]. First, a hydrogen atom removes from the side chain of polyunsaturated fatty acids, forming the lipid radical. Then the unpaired electron rearranges, forming conjugated dienes. And the lipid radical converts into lipid peroxyl radical by attracting molecular oxygen. Second, the lipid peroxyl radical extracts a hydrogen atom and begins a cycle of peroxidation reaction. Third, two radicals combine and form a nonradical. Beside hydroperoxides, lipid peroxidation also produces aldehydes, lipid hydroxides, and others. The lipid peroxidation of n-3 PUFAs is a complex process and ultimately produces the 4-hydroxynonenal. The peroxidation of n-3 PUFAs majorly produces the 4-hydroxy-2E-hexenal (4-HHE); the 4-hydroxy-2E-nonenal (4-HNE) is the major product of n-6 PUFAs, and some other fatty acids produce 4-hydroxy-2E, 6Z-dodecadienal (4-HDDE) [25]. The lipid peroxidation will accumulate these products and affect the normal cell functions, leading to cell death at last. The peroxidation of n-3 PUFAs belongs to nonenzymatic lipid peroxidation, which derived from free radical reactions [26]. The reaction between ROS and transition metals produce the hydroxyl radical, the major radical in this process. The lipid peroxidation will indicate the overproduction of ROS, and this vicious circle may cause the increase of ROS, necrosis, and apoptosis during the time [27]. In addition, the generation of excessive ROS reduces the activation and bioavailability of NO [28]. Oxidative stress and ROS are detrimental factors in the progression of cerebral I/R injury [29]. Oxidative stress can increase the expression of cytokine and the occurrence of edema and apoptosis [30]. During reperfusion, ROS acts as the signaling molecules, inducing the activation of NF-*κ*B and activator protein-1 (AP-1) [31]. Due to the low activities of antioxidant enzymes and the high rates of oxidative metabolic activities, neurons in the brain are more vulnerable to ischemic damage [32]. Free radical generation, calcium overload, excitatory neurotransmitter accumulation, inflammation, and apoptosis are all related to neuronal injuries after ischemic damage [33, 34].

**Figure 2:**
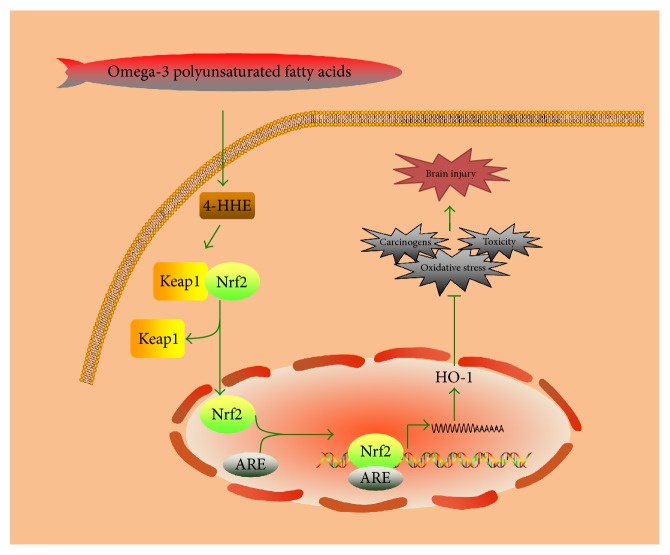
The Nrf2/HO-1 signaling pathway induced by n-3 PUFAs.

The dietary supplementation of n-3 PUFAs can decrease the volume of cerebral infarction partly by adjusting antioxidant enzymes activities and partly by working as an antioxidant directly [35]. n-3 PUFAs may act as an antioxidant in reducing cerebral lipid peroxides and play a role in regulating oxidative stress through the increase of oxidative burden and the improvement of antioxidative defense capacity [36]. The chronic administration and dietary supplementation of n-3 PUFAs can improve symptoms of cerebral I/R by increasing the antioxidative capacity, as well as reducing the induction of chaperon molecules and the stabilization of membrane integrity and lipid peroxidation [37]. The dietary supplementation of ALA is also found such that it can reduce the level of lipid peroxidation, as well as increasing the risk of spontaneous reperfusion [38]. The neuroprotective effects of n-3 PUFAs include not only inhibiting the oxidative stress but also enhancing the expression of nuclear factor E2-related factor 2 (Nrf2)/heme oxygenase-1 (HO-1) [39]. The Nrf2/HO-1 signaling pathway induced by n-3 PUFAs is shown in Figure 2. Nrf2/HO-1 signaling pathway is a crucial mechanism of n-3 PUFAs for protecting cells [40]. n-3 PUFAs reduce ischemic injury by activating Nrf2 and increasing HO-1 production [41]. The protective mechanisms are associated with the upregulation of HO-1, the activation of Nrf2, and the oxidation of 4-HHE. 4-HHE is the end-product of n-3 PUFAs by peroxidation and acts as an effective Nrf2 inducer [42]. Nrf2 acts as a transcription factor in regulating phase-2 enzymes expression. Under normal conditions, Kelch-like ECH-associated protein-1 (Keap1), the inhibitory protein of Nrf2, will bind to Nrf2 and lead Nrf2 to the proteasomal degradation process. Under oxidative stress, 4-HHE will react with the cysteine residues of Keap1 and dissociate Nrf2 from Keap1 [41]. Then Nrf2 will translocate into the nucleus, bind to antioxidant responsive element (ARE), and induce the expression of phase-2 enzymes [43]. Phase-2 enzymes like HO-1 mainly mediate the cytoprotection against oxidative stress, carcinogens, and toxicity. Cerebral ischemia/reperfusion injury will cause the elevated expression of antioxidative proteins and Keap1/Nrf2 system and increase the expression of Nrf2 and HO-1, which can inhibit the activation of microglia and the expression of proinflammatory cytokine [44]. The shortage of Nrf2 or HO-1 will sensitize animals to inflammation and injury induced by ischemic stroke. The neuroprotection against cerebral ischemic stroke mediated by DHA includes improving neuronal defense capacity and inhibiting cellular inflammatory mechanisms by increasing the expression of Nrf2 and HO-1 [45]. Although DHA itself is able to increase the expression of Nrf2 and HO-1 in glial cell cultures, it is not enough to induce the promotion of Nrf2 and HO-1 in vivo. Actually, the treatment with DHA after ischemic stroke only can provide a driving force for the promotion of Nrf2 and HO-1 [46].

On the other side, n-3 PUFAs exhibit the modulatory effects on the homeostasis of redox potential by enhancing the oxidative burden induced by lipid peroxidation and activating the activity of antioxidant enzymes [47]. Under normal conditions, n-3 PUFAs such as DHA may not act as initiators of free radical generation, while in the oxidized environments DHA may augment the oxidative burden. That is to say, n-3 PUFAs treatments may lead to augmenting, or be comparable at least to, the damage induced by cerebral I/R injury [48]. People found that DHA could increase the activity of MPO, the expression of COX-2 mRNA, and the activity of caspase 3, which will exacerbate neurobehavioral deficits and cerebral infarction in the end [49]. The acute posttreatments with DHA after cerebral ischemic stroke are found to augment oxidative burden and subsequently exacerbate cerebral I/R injury remarkably [37]. The detrimental effects of DHA in cerebral I/R are associated with oxidative changes. DHA alone has little effects on the generation of free radical in neuroglia but increases the oxidative burden induced by hydrogen peroxide greatly. The high level of free n-3 PUFAs will induce free radicals to react with unsaturated fatty acids instantaneously [37]. Once beginning, the reaction will continue and propagate an amplification cycle of free radicals generation, which will lead to the augmented oxidative stress and increase the oxidative burden induced by cerebral I/R significantly.

In terms of inflammation, ischemic stroke will trigger complex cellular responses, including the recruitment of inflammatory cells and the activation of glial cells [50]. Leukocytes will move in the interstitial compartments and release the proteolytic enzymes and cytotoxic metabolites, inducing the nerve cells death and enhancing the deleterious effects of ischemic stroke. In the end, leukocytes plugging in capillaries, the aggregation of platelet leukocyte, and the extravasation of albumin occur [51]. n-3 PUFAs are found to inhibit systemic inflammatory responses and modulate vascular inflammation by changing intracellular signal transduction and controlling lipid mediators [52]. The anti-inflammatory effects of n-3 PUFAs include inhibiting the conversion of arachidonate acids to the proinflammatory lipid intermediates, interrupting the NF-*κ*B signaling pathway, and activating the AMP-activated protein kinase, inducing the synthesis of anti-inflammatory lipid mediators like resolvins and protectins [40]. DHA is the precursor of neuroprotectin D1 (NPD1) and NPD1 can downregulate apoptosis, promote neurogenesis, and inhibit leukocyte infiltration and the expression of proinflammatory gene [53]. n-3 PUFAs also exhibit potent immunomodulatory effects by reducing the leukocyte chemotaxis and inhibiting the expression of adhesion molecules [54]. EPA and DHA can exhibit neuroprotective effects through inducing the expression of receptors of chemoattractants and inhibiting the activation of macrophages and microglia and the migration of neutrophils and monocytes. DHA also can increase the generation of antiapoptotic proteins such as Bcl-xL and Bcl-2, which inhibit the inflammatory response mediated by microglial cells [55]. In glial cell culture, DHA exhibits immunosuppressive effects by reducing the phosphorylation of c-Jun N-terminal kinase (JNK) and c-Jun and inhibiting the activation of AP-1 [56]. The activation of JNK plays a crucial role in neuroinflammation and cell death induced by ischemic stroke [57]. Once activated, JNK will increase the phosphorylation of c-Jun, the crucial component of AP-1, and induce the cell death program and transcription-dependent inflammation [58]. The downregulation of JNK/AP-1 signaling pathway, which includes decreasing the phosphorylation of c-Jun and JNK and inhibiting the DNA-binding activity of AP-1, contributes to the neuroprotective effects of DHA against cerebral ischemic stroke [59]. Someone found that G protein-coupled receptor 120 (GPR120) could be activated by long-chain fatty acids and GPR120 acted as a functional receptor or sensor of n-3 PUFAs, exerting the anti-inflammatory effects [60]. Through GPR120, n-3 PUFAs inhibit the activation and phosphorylation of TAK1 by the *β*-arrestin2/TAB1 dependent effect, resulting in the inhibition of TNF-a and TLR inflammatory signaling pathways [61].

### 4.3. n-3 PUFAs and Hemorrhagic Stroke

SAH is a pathologic syndrome defined by the appearance of the blood in the subarachnoid space resulting from a wide variety of causes. The most common cause of SAH is trauma, and 85% of nontraumatic patients are in case of underlying cerebral aneurysm [62, 63]; the other 15% are idiopathic [64]. Two-thirds of the idiopathic patients are due to perimesencephalic hemorrhage [65, 66]. According to unenhanced CT, SAH is classified into mainly three distinct forms [67]. Aneurysm rupture and vascular malformation belong to the first form, in which SAH is centered in the central basal or suprasellar cisterns and extends to periphery diffusely [68]. Idiopathic perimesencephalic hemorrhage resulting from aneurysm rupture, vascular malformation, and cervicomedullary junction tumor belong to the second form, in which SAH is centered in the low basal or perimesencephalic cisterns and does not extend. The third form, in which SAH is centered in the cerebral convexities, includes cerebral amyloid angiopathy, reversible cerebral vasoconstriction syndrome, cerebral venous thrombosis, and posterior reversible encephalopathy syndrome. There are several reasons behind the morbidity and mortality of the patients with SAH and cerebral vasospasm (CV) is a significant one of them [69]. The pathogenesis of cerebral vasospasm is still unclear. Inflammation, Endothelin (ET), NO, and products of erythrocyte degradation all have been confirmed to play crucial roles in CV [70, 71]. OxyHb, produced by erythrocyte degradation, is one of the causes of CV. When blood flows into the subarachnoid space and soak vessels for a long time, dysfunction of vessels occurs and then the blood cells begin to collapse and lipid peroxide and free radicals produced [72]. The products lead to a series of chain reactions, the destruction of biological membrane, the removal of endogenous NO, and the increasing production of ET. The diastolic and systolic function of vessels failed at last, leading to cerebral hemorrhage [73]. Quite a few studies are indicative of the fact that Rho-kinase plays a crucial role in CV [74, 75], and some agents like thromboxane A2 (TXA2) and sphingosylphosphorylcholine (SPC) can activate Rho-kinase [76, 77]. Recently, EPA is reported to inhibit SPC by inducing the activation of Rho-kinase in vitro [78]. Moreover, EPA can change the concentration of arachidonic acid, which has a potential role in CV [79] and inhibit the synthesis of TXA2 [80]. The concentration of free fatty acids increases after SAH and has a secondary elevation between 8 and 10 days after SAH [79]. These observations are suggestive of the fact that EPA can inhibit CV after SAH and improve clinical prognosis by inhibiting the activation of Rho-kinase [81]. Furthermore, oral EPA is found to reduce the risk of CV after SAH [82], and using n-3 PUFAs for immunomodulatory interventions could reduce the risk of delayed cerebral ischemia after SAH [83].

ICH is a pathologic syndrome caused by the rupture of intracranial vessel, which is resulting from nontraumatic factors, and the appearance of the blood in the intracerebral space leads to several causes. Because n-3 PUFAs demonstrate poor effects on ICH, there are a few studies focusing on n-3 PUFAs and ICH. A slice of studies suggests that different concentration of n-3 PUFAs leads to different effects [84]. Low levels of n-3 PUFAs protect against thrombogenesis, while high levels may induce oxidative damage and become a risk factor for ICH [85]. Moreover, high levels may lead to a poor functional outcome and a severe motor impairment after ICH [86].

## 5. Expectation

Except the antioxidant and anti-inflammatory effects, n-3 PUFAs also can trigger other responses like neuranagenesis and revascularization in stroke. The classification of fatty acids is shown in Figure 1, and the Nrf2/HO-1 signaling pathway induced by n-3 PUFAs is shown in Figure 2. Even though n-3 PUFAs is generally accepted as a beneficial factor in diets, there are still many debates remaining. As most of the previous studies focus on the prevention and post-treatments of stroke, the lack of systematic treatment strategy with n-3 PUFAs remained to be supplemented.

## Abbreviations

n-3 PUFA: Omega-3 polyunsaturated fatty acids
N-6 PUFA: Omega-6 polyunsaturated fatty acids
DHA: Docosahexaenoic acid
EPA: Eicosapentaenoic acid
ALA: Alpha-linolenic acid
TIA: Transient ischemic attack
SAH: Subarachnoid hemorrhage
ICH: Intracerebral hemorrhage
CBF: Cerebral blood flow
I/R: Ischemia/reperfusion
ROS: Reactive oxygen species
4-HHE: 4-Hydroxy-2E-hexenal
4-HNE: 4-Hydroxy-2E-nonenal
4-HDDE: 4-Hydroxy-2E, 6Z-dodecadienal
AP-1: Activator protein-1
Nrf2: Nuclear factor E2-related factor
HO-1: Heme oxygenase-1
Keap1: Kelch-like ECH-associated protein-1
ARE: Antioxidant responsive element
NPD1: Neuroprotectin D1
JNK: c-Jun N-terminal kinase
GPR120: G protein-coupled receptor 120
CV: Cerebral vasospasm
ET: Endothelin
TXA2: Thromboxane A2
SPC: Sphingosylphosphorylcholine.
